# Delivery of the gene encoding the tumor suppressor Sef into prostate tumors by therapeutic-ultrasound inhibits both tumor angiogenesis and growth

**DOI:** 10.1038/s41598-017-12408-1

**Published:** 2017-11-08

**Authors:** Sabrin Mishel, Boris Shneyer, Lina Korsensky, Orit Goldshmidt-Tran, Tom Haber, Marcelle Machluf, Dina Ron

**Affiliations:** 1 Department of Biology, Technion, Israel Institute of Technology, Haifa, Israel; 2 Department of Biotechnology and Food Engineering, Technion, Israel Institute of Technology, Haifa, Israel; 3 Present Address: Department of Molecular Medicine, Beckman Research Institute, City of Hope, Duarte, CA USA; 4 Present Address: Department of Immunology, Faculty of Medicine, University of Toronto, Program in Genetics and Genome Biology, The Hospital of Sick Children, Toronto, Canada

## Abstract

Carcinomas constitute over 80% of all human cancer types with no effective therapy for metastatic disease. Here, we demonstrate, for the first time, the efficacy of therapeutic-ultrasound (TUS) to deliver a human tumor suppressor gene, hSef-b, to prostate tumors *in vivo*. Sef is downregulated in various human carcinomas, in a manner correlating with tumor aggressiveness. *In vitro*, hSef-b inhibited proliferation of TRAMP C2 cells and attenuated activation of ERK/MAPK and the master transcription factor NF-κB in response to FGF and IL-1/TNF, respectively. *In vivo*, transfection efficiency of a plasmid co-expressing hSef-b/eGFP into TRAMP C2 tumors was 14.7 ± 2.5% following a single TUS application. Repeated TUS treatments with hSef-b plasmid, significantly suppressed prostate tumor growth (60%) through inhibition of cell proliferation (60%), and reduction in blood vessel density (56%). In accordance, repeated TUS-treatments with hSef-b significantly inhibited *in vivo* expression of FGF2 and MMP-9. FGF2 is a known mitogen, and both FGF2/MMP-9 are proangiogenic factors. Taken together our results strongly suggest that hSef-b acts in a cell autonomous as well as non-cell autonomous manner. Moreover, the study demonstrates the efficacy of non-viral TUS-based hSef-b gene delivery approach for the treatment of prostate cancer tumors, and possibly other carcinomas where Sef is downregulated.

## Introduction

Prostate cancer (PCa) is the second deadliest cancer in the western world^1^. While radical treatment of organ-confined PCa can improve survival, few therapeutic options are available for hormone refractory and metastatic prostate cancer. The fibroblast growth factor (FGF) axis and the transcription factor, NF-κB, have been implicated in prostate carcinogenesis^2–4^ and both are considered potential targets for therapeutic intervention. FGFs signal via four distinct high-affinity cell-surface tyrosine kinase receptors, designated FGFR1–FGFR4^5^. Gain- and loss-of-function studies in mouse models have demonstrated the requirement for the FGF signaling axis in prostate development and homoeostasis^6^. Expression of some members of the FGF family, such as FGF8 and FGFR4, is significantly elevated in clinical prostate cancer^4^, and high expression levels of FGFR4 are associated with metastatic disease^7^. NF-κB proteins are an important class of transcriptional regulators in PCa. Their overactivation correlates with PCa chemoresistance, advanced disease stage and prostate-specific antigen (PSA) recurrence. Activation of NF-κB signaling promotes castrate-resistant growth of PCa [reviewed in^8^]. Additionally, NF-κB signaling is upregulated in a subset of castrate-resistant prostate cancer patients and correlates with disease progression^9^.

Sef (IL-17RD) is a tumor suppressor that is highly conserved in vertebrates. The human Sef gene (designated hSef) encodes various isoforms which are generated via alternative splicing, including the hSef-a and the hSef-b isoforms^10,11^. The hSef-a isoform encodes for a receptor-like glycoprotein, and is a feedback antagonist of FGF signaling^12–16^. Recently, we discovered that hSef-a can also antagonize pro-inflammatory cytokine signaling through cytoplasmic sequestration of NF-κB^17^. When overexpressed in a PCa cell line, hSef-a retarded their growth in an *in vivo* xenograft model^15^. Unlike hSef-a, the hSef-b isoform encodes a cytosolic protein which is translated from an alternative start site (CUG). Non-AUG codons direct less efficient translation initiation^18,19^. Hence, when translated *in vitro* or expressed in cells under the control of the same promoter, the hSef-b protein is expressed at significantly lower levels as compared to hSef-a^10,14^. In spite of its lower expression levels, hSef-b inhibits FGF-mediated mitogenic activity as potently as hSef-a indicating that hSef-b “specific activity” is higher^10,14^. This provides a strong impetus for studying the therapeutic potential of the “b” isoform. Thus far, nothing is known about the effect of hSef-b on tumor growth, and whether it is capable of inhibiting pro-inflammatory cytokine signaling.

Sef status in cancer has been studied by several groups including our own. It was found that Sef expression is downregulated in essentially every carcinoma type examined thus far including breast, thyroid, ovarian, colon and prostate cancers, in a manner correlating with tumor aggressiveness^20–22^. In prostate cancer, FGFR4 overexpression combined with hSef downregulation predicts the development of metastasis and thus poor prognosis^7^. Silencing hSef expression in a PCa cell line enhanced serum-dependent migration/invasion *in vitro* and *in vivo* ^15,20,23^, and accelerated FGF and interleukin-1 (IL-1) dependent cell proliferation in a cervical carcinoma cell line^14,21^. Collectively, hSef properties make it an attractive candidate for cancer gene therapy.

In the current study, we evaluated the potential of hSef-b for cancer gene therapy using a delivery approach that is based on therapeutic ultrasound waves (TUS). Ultrasound is a non-viral approach for non-invasive delivery of genes into cells and tissues^24–27^. Among the ultrasound modalities approved for clinical application, TUS, which operates at frequencies of 1–3 MHz and utilizes relatively low intensities (0.1–2 W/cm^2^), is considered a promising technology for *in vivo* transfection^25^. Previously we demonstrated that TUS-mediated delivery of a gene encoding for PEX, an inhibitor of angiogenesis, significantly repressed tumor angiogenesis with no toxicity^28^. Here, we demonstrate that even a single TUS application can lead to the delivery of the cDNA encoding for hSef-b into TRAMP C2 prostate tumors inoculated in mice. Our studies reveal that such direct TUS transfection of hSef-b plasmid DNA and its subsequent expression effectively suppress TRAMP C2 tumor growth *in vivo*. Importantly, hSef-b inhibited not only tumor cell proliferation but also tumor angiogenesis, a previously unknown Sef function. In cultured TRAMP C2 cells, hSef-b suppressed both FGF-induced ERK/MAPK activation and cytokine- induced activation of NF-κB.

## Results

### hSef-b inhibits TRAMP C2 growth *in vitro*

**Figure 1:**
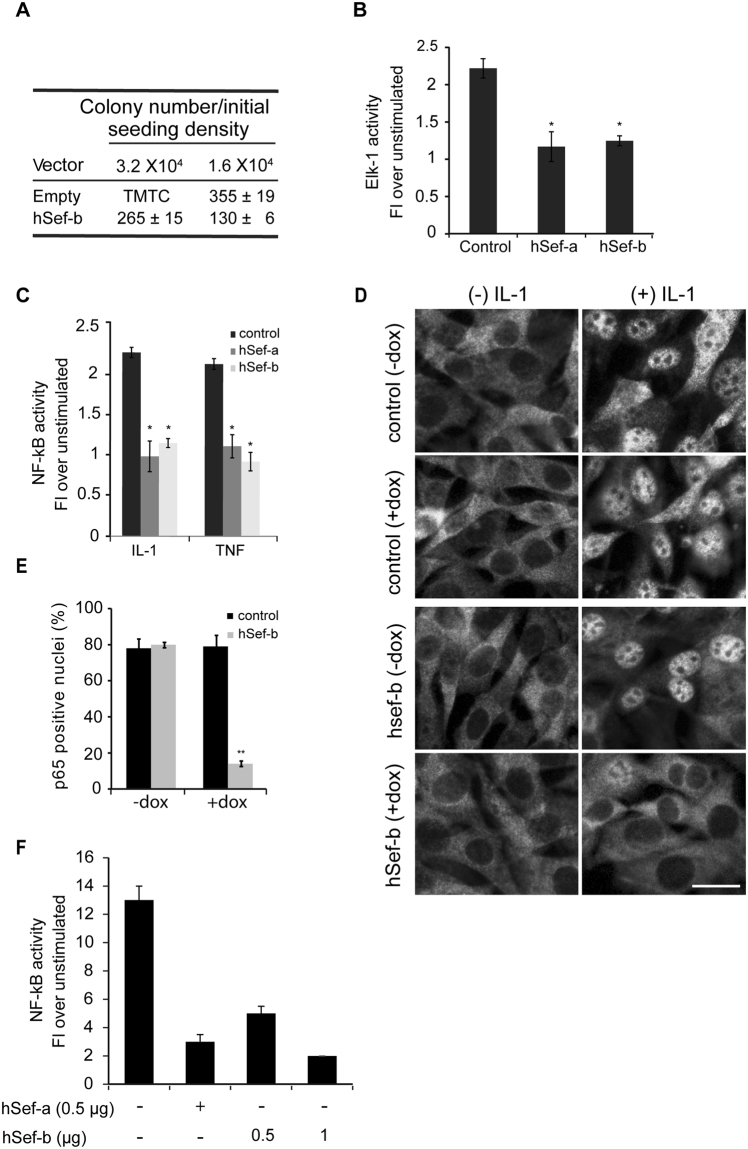
(**A**) hSef-b suppresses colony formation in TRAMP C2 cells. Cells were stably transfected with 5 µg hSef-b (pCDNA/hSef-b) or an empty vector (pCDNA) along with eGFP construct (0.1 µg) for monitoring transfection efficiency. After one day, transfection efficiencies were microscopically monitored, cells were seeded at different densities and selected with G418 for ~2 weeks. Clones were counted at the end of the selection process. The results are normalized to transfection efficiencies, and are representative of 2 independent experiments. TMTC denotes: too many to count. (**B**,**C**) hSef isoforms suppress ERK/MAPK and NF-κB in TRAMP C2 cells. Cells were transfected with Elk-1 or NF-κB- luciferase reporter plasmid along with a control empty vector or with hSef-a or hSef-b expression vector. Cells were treated with FGF2 (2.5 ng/ml) for inducing Elk-1 activation, and with IL-1 or TNF (5 ng/ml) for inducing NF-kB activation. Error bars indicate SEM (N = 2, * p < 0.05). (**D**,**E**) hSef-b attenuates IL-1 induced NF-κB (p65) nuclear translocation in TRAMP C2 cells. Control or hSef-b Tet on/TRAMP C2 cells were grown in the absence or presence of dox for 24 hr, then stimulated for 15 minutes with 5 ng/ml IL-1 and immunostained with α-p65 antibody. Representative images (**D**) were quantified for p65 nuclear localization [E, (N = 2, ** p ≤ 0.004)]. More than 300 cells from each sample were microscopically examined. Bar: 10 µm. (**F**) hSef-b inhibits cytokine induced NF-κB activation in human cervical carcinoma cells. HeLa cells were transfected with NF-κB luciferase reporter plasmid along with each empty vector (0.5 µg/ml), hSef-a or hSef-b constructs at the indicated concentrations. The assay was performed following stimulation with 5 ng/ml IL-1. FI denotes: Fold Increase.

To examine the general effect of hSef-b on the growth of TRAMP C2 cells, we performed a colony assay. TRAMP C2 cells were stably transfected with an expression vector bearing hSef-b (pCDNA3.1/hSef-b) or the empty vector along with enhanced green fluorescent protein (eGFP) for monitoring transfection efficiency. One day later, cells were plated at different seeding densities and marker selected for about 2 weeks. Cells transfected with the hSef-b expression vector formed ~2.7 fold less colonies when compared to cells transfected with the control vector (Fig. 1A). Since hSef-b does not promote apoptosis^10,14^, the observed colony suppression most likely results from inhibition of TRAMP C2 cell proliferation.

### hSef-b inhibits both FGF and pro-inflammatory cytokine signaling in TRAMP C2 cells

hSef-b inhibits FGF dependent proliferation of fibroblasts via attenuation of ERK/MAPK^10^, the classical kinase controlling cell proliferation^29^. We therefore examined the effect of the hSef-b isoform on FGF-induced ERK/MAPK activation in TRAMP C2 cells using Elk-1 luciferase reporter assay. The Elk-1 transcription factor is a downstream target of activated ERK/MAPK^30^. TRAMP C2 cells were transfected with an Elk-1 reporter plasmid alone or with a plasmid encoding either hSef-b or hSef-a which served as a positive control. Cells were, then, stimulated with FGF-2 (2.5 ng/ml). Transfection of TRAMP C2 cells with 0.5 µg hSef-b expression vector was sufficient to inhibit FGF-2 induced Elk-1-dependent reporter activity by 44% ± 2% [Fig. 1B, (p < 0.05)]. The hSef-b inhibitory potency was comparable to that of hSef-a despite the known lower translation efficiency of the hSef-b protein as compared to hSef-a^10,14^.

Given the role of NF-κB in PCa, and our previous findings that hSef-a can inhibit NF-κB activation in response to pro-inflammatory cytokines^17^, we next examined whether hSef-b can also inhibit NF-κB in TRAMP C2 cells using hSef-a as a positive control for NF-κB inhibition. The hSef-b isoform, similar to hSef-a, effectively inhibited NF-kB reporter activity in response to IL-1 as well as in response to tumor necrosis factor (TNF) by 55% ± 4% and 64% ± 5%, respectively [p < 0.05, (Fig. 1C)]. To find out whether the effect of hSef-b on NF-κB activation results from attenuated NF- κB nuclear translocation, we utilized TRAMP C2 stable cell-lines in which hSef-b expression is regulated in an inducible manner (Tet on/TRAMP C2). Control and TRAMP/hSef-b cells, grown in the absence or presence of doxycycline (dox) for 24 hrs, were stimulated with IL-1 for 15 minutes and then endogenous p65 was visualized by indirect immunofluorescence (IF). Nuclear translocation of p65 in un-induced TRAMP/hSef-b cells was similar to that observed in the control cultures grown with or without dox (79% ± 5%). By contrast, p65 nuclear translocation was reduced by 5.7 fold (p ≤ 0.004) in TRAMP/hSef-b cells grown in the presence of dox (Fig. 1D and E). To further examine whether hSef-b can also inhibit pro-inflammatory cytokine signaling in other carcinoma types, we tested its effect on IL-1 induced NF-κB activation in a human cervical carcinoma cell line (Hela cells). We found that 0.5 µg of transfected hSef-b plasmid attenuated NF-κB activation by 63%, and increasing plasmid amount to 1 µg markedly inhibited NF-κB (86% inhibition) in Hela cells (Fig. 1F). Collectively, the above described results indicate that hSef-b is capable of inhibiting two signaling networks implicated in PCa progression.

### Efficiency and kinetics of gene expression following a single TUS application *in vivo*

**Figure 2:**
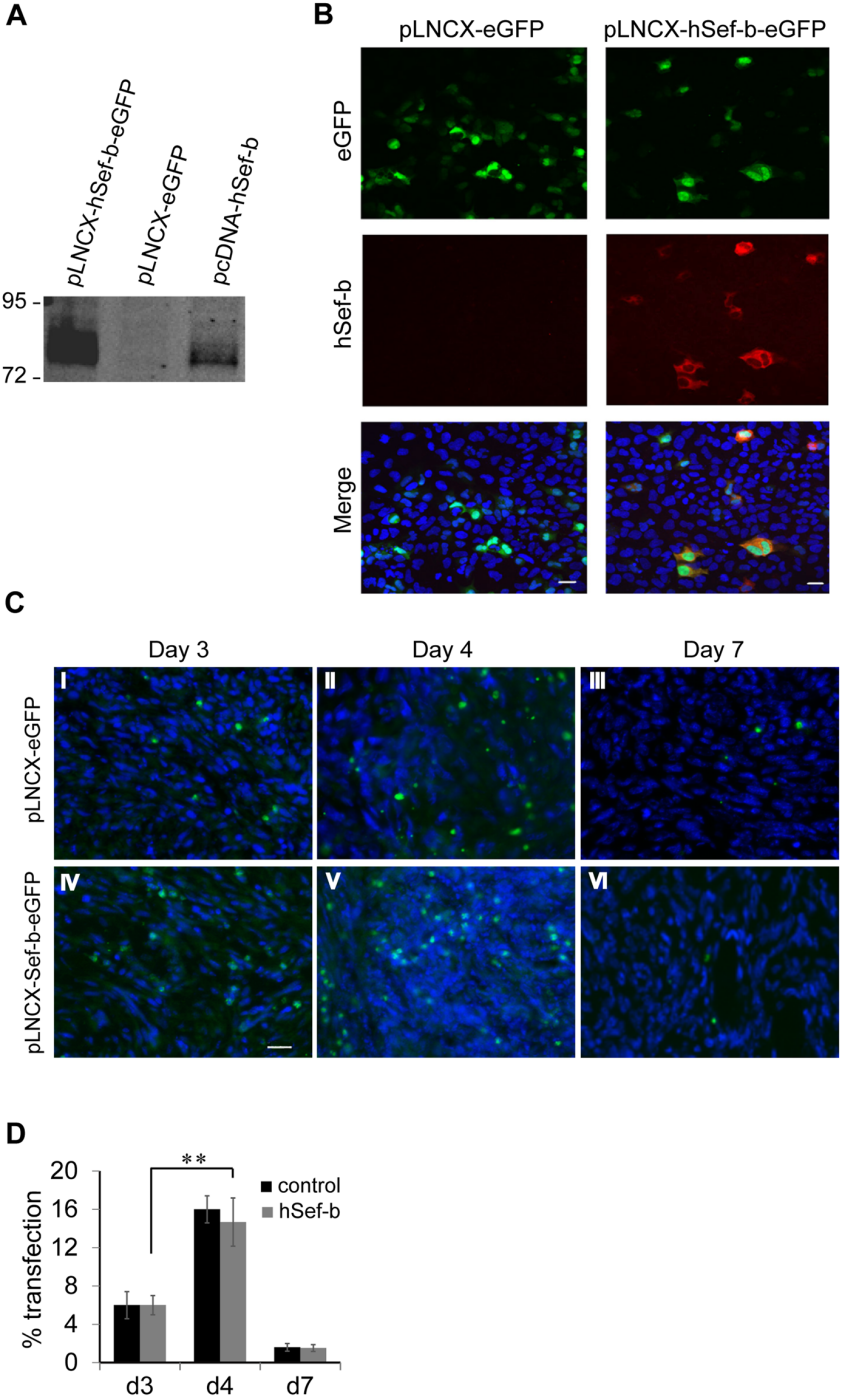
(**A**,**B**) Co-expression of hSef-b/eGFP *in vitro*. HEK 293 cells were transfected with pLNCX-eGFP, bicistronic pLNCX-hSef-b/eGFP vector or pcDNA3.1/hSef-b. Whole cell lysates were subjected to Western blot analysis with antibody directed against myc-epitope tag that is fused to hSef-b protein (**A**). Cellular expression of hSef-b was analyzed by IF using anti-myc antibody (red) and standard fluorescence microscopy. Bar: 10 μm (**B**). (**C**) Kinetics of expression of eGFP reporter *in vivo*. Subcutaneous tumors were injected with control pLNCX-eGFP vector (I–III) or pLNCX-hSef-b/eGFP vector (IV–VI) followed by a single TUS application. Micrographs are representative of 3 sections taken from each mouse (N = 5). Tumor sections (20 µm) from day 3, 4 and 7 post DNA transfection were examined by fluorescent microscopy. Bar: 20 µm. (**D**) Quantification of transfection efficiencies *in vivo*. Micrographs are representatives of three sections taken from each mouse, five mice in each group. Transfection efficiencies were calculated using LUCIA Image Analyses (**P ≤ 0.01)

Having established that hSef-b negatively regulates FGF and pro-inflammatory cytokine signaling in TRAMP C2 cells, we next aimed at investigating whether hSef-b gene delivery into TRAMP C2 tumors can suppress their growth *in vivo*. To readily monitor the efficiency of transfection *in vivo*, we have generated a bicistronic expression vector for the expression of hSef-b and IRES-linked eGFP reporter. To find out if eGFP can indeed serve as a reporter for hSef-b expression, we examined the expression of both proteins following transient transfection of the hSef-b/eGFP expression construct in HEK 293 cells. The hSef-b protein was efficiently translated as determined by immunoblotting of whole cell extracts and IF staining (Fig. 2A and B), and both hSef-b/eGFP proteins were co-expressed in the majority of transfected HEK 293 cells (Fig. 2B). These findings established that eGFP can serve as a reporter for hSef-b protein expression.

Next, we employed a single TUS treatment to assess the efficiency of hSef-b/eGFP plasmid delivery and expression kinetics *in vivo*. Four-five ﻿weeks old C57 black 6 (C57BL/6) male mice (N = 12) were inoculated subcutaneously (s.c.) in the flank with 2 × 10^6^ TRAMP C2 cells per mouse. When tumors reached a palpable size (~100 mm^3^), animals were injected intra-tumorally (i.t.) with 100 µg of the hSefb/eGFP construct. Then, a single TUS treatment was applied on the skin area above the tumor using previously described conditions^26^. Since previous studies indicated that transgene expression is barely detectable prior to day 3 of DNA delivery^31^, we began monitoring eGFP expression from day 3 post DNA transfection. Mice were sacrificed 3, 4, and 7 days post DNA delivery, and tumor sections were examined microscopically for distribution of eGFP expression. eGFP expression was detectable on day 3 of DNA administration (about 6% positive tumor cells transfected with either the control or the hSef-b construct). Transfection peaked at day 4 where about 15–16% of cells transfected with hSef-b or control construct were positive for eGFP fluorescence, (Fig. 2C, and data not shown), an efficiency that is slightly better than previously published transfection efficiency of a reporter gene into mouse or human PCa tumors following TUS without the use of contrast agent^28^. Reporter expression declined on day 7 to less than 2%, in parallel with the decrease in the levels of the injected DNA in the tumors (Fig. 2C, data not shown).

### The effect of *in vivo* expression of hSef-b on tumor growth

**Figure 3:**
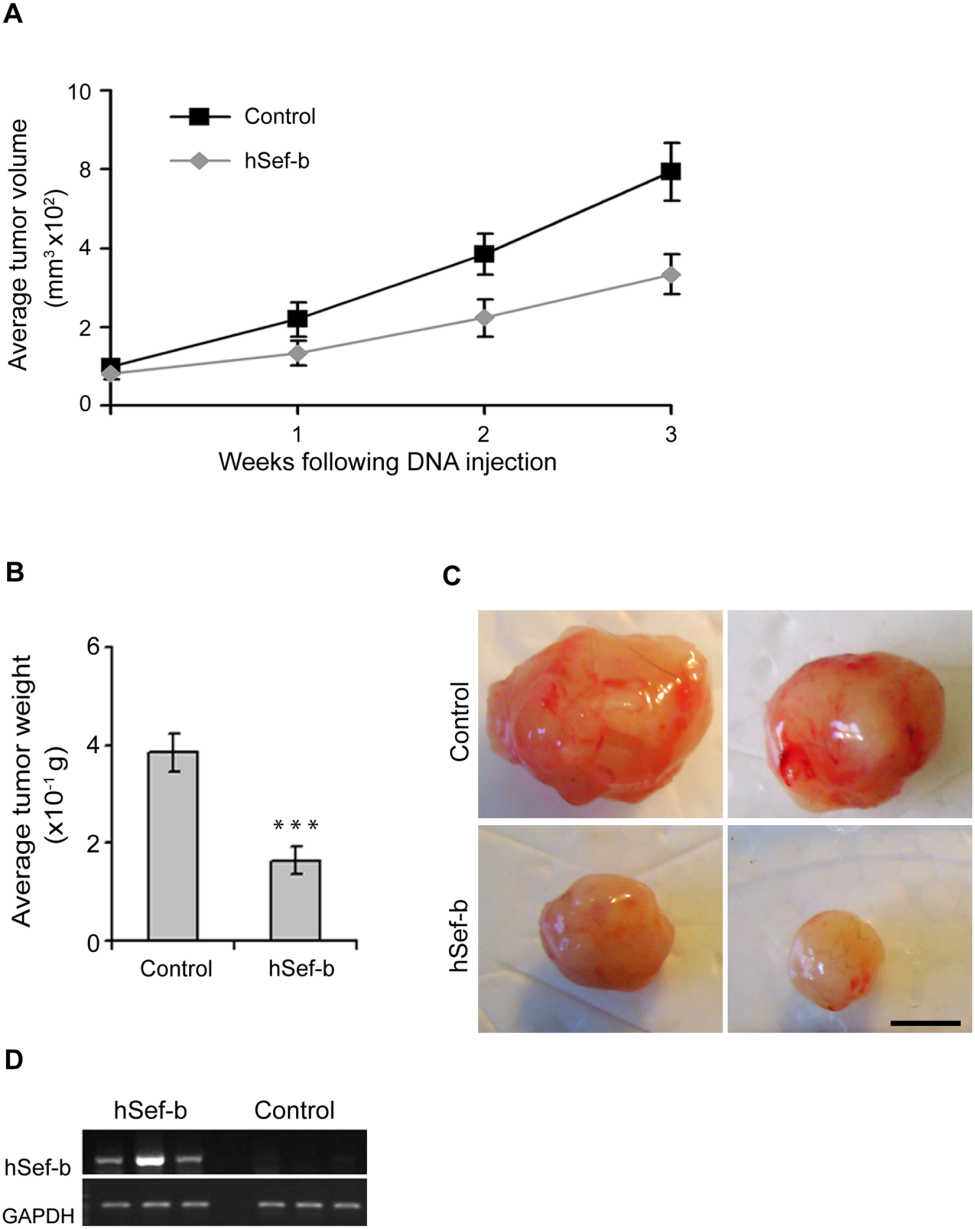
hSef-b suppresses tumor growth *in vivo*. (**A**) Average volume of tumors transfected with control empty vector (N = 14) or with hSef-b construct (N = 12) by TUS. Tumor volume was measured at weekly intervals during the 3 rounds of DNA injections. (**B**) Average tumor weight at the end of the experiments (***p ≤ 0.0001). (**C**) Images of representative tumors. Bar: 2.5 mm. **(D**) RT-PCR analysis for the detection of hSef-b transcript in total RNA extracted from the resected tumors. Amplification was carried out with hSef-b specific primers.

Based on the results obtained from the single treatment, additional *in vivo* experiments were conducted in order to test the effect of hSef-b on tumor growth *in vivo*. Mice inoculated with TRAMP C2 cells, were subjected to repeated treatments of TUS with control or hSef-b plasmids (N = 15 in each group). DNA transfection and TUS application were carried out once a week for 3 weeks. In each experiment mice bearing the TRAMP C2 tumors were randomly divided into two groups, one group receiving the control plasmid and the other the hSef-b plasmid. In the initial experiment we also tested the effect of TUS alone on tumor growth as compared to untreated tumors (N = 5 mice in each group). Subcutaneous tumor growth was measured with a caliper twice a week for the entire period of each experiment (21 days), and tumor volume was calculated. All mice were sacrificed after 21 days, tumors were excised, photographed (representative images are shown in Fig. 3C) and their weight was measured. In agreement with our previously published data^28^, TUS alone or TUS applied on tumors transfected with the control empty-vector did not facilitate tumor growth as compared to untreated tumors (Fig. S1). By contrast, tumor growth was markedly inhibited in tumors transfected with the hSef-b plasmid as compared with the control group [60% inhibition; p ≤ 0.0001) Fig. 3, panels A–C]. Expression of hSef-b from the transfected plasmid was evaluated by RT-PCR with primers specific to human Sef transcripts. RNA was extracted from a small tumor portion (N = 3 for each control and hSef-b group) 7 days post last DNA transfection. Although plasmid levels decline significantly at day seven, hSef-b mRNA expression could be readily observed to various degrees in all 3 tumors injected with the hSef-b plasmid but not in the control tumors (Fig. 3D). The variability in hSef-b mRNA expression may reflect differences in DNA transfection efficiencies, or site specific transfection as the RNA was prepared from a small tumor portion. Nevertheless, these results clearly indicate that repeatedly transfected hSef-b DNA into pre-established PCa tumors can effectively suppress their growth *in vivo*.

### The effect of hSef-b on tumor tissue morphology, PCa cell proliferation *in vivo*, and tumor angiogenesis

**Figure 4:**
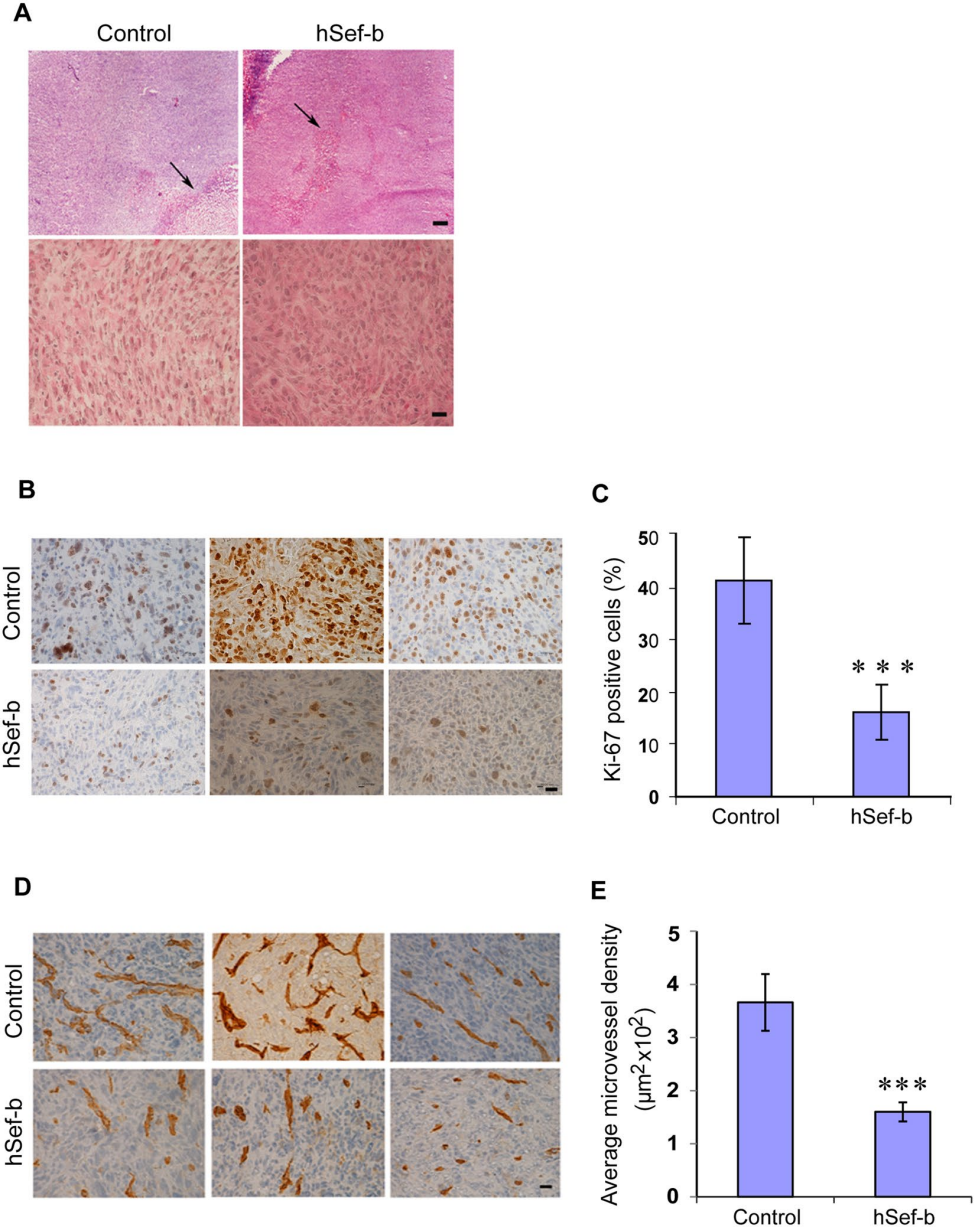
The effect of hSef-b on tumor cell proliferation and tumor angiogenesis *in vivo*. (**A**) Tumors were resected 7 days post 3^rd^ TUS application. Tumor tissue sections were stained with Hematoxylin and Eosin for evaluation of tumor morphology. Micrographs are representative of at least 3 sections from each tumor (14 and 12 tumors transfected with the control or the hSef-b plasmid, respectively). Arrows point to regions of necrosis. Bars: 200 and 20 μm for the upper and lower bar, respectively. (**B**) Tumors transfected using TUS, with either the control or hSef-b constructs, were harvested 21 days post first DNA injection. IHC was carried out with an antibody against mouse Ki-67 on 7 µm frozen sections. Bar: 20 µm. (**C**) Quantification of the percentage of Ki-67 positive cells (indicative of % of proliferating cells,***p ≤ 0.0001). (**D**) IHC was carried out with an antibody against mouse CD31 on 7 µm frozen sections. Bar: 20 µm. (**E**) Quantification of microvessel density (***p ≤ 0.0016). A total of 9 sections were analyzed from 3 individual tumors in each group for both ki67 and CD31 immunostaining.

Frozen sections of control and hSef-b tumors were stained with H&E to examine their tissue morphology. As shown in Fig. 4, the tumor morphology was mostly preserved except for necrotic regions located at the center of the tumor tissue (Fig. 4A). To examine whether hSef-b inhibition of tumor growth *in vivo* correlates with reduced proliferation rate, we evaluated the expression level of the nuclear protein Ki-67, a marker for cell proliferation, by immunohistochemistry (IHC). Microscopic examination of sections from different tumor regions of mice that received hSef-b DNA (N = 3) and from control tumors (N = 3) clearly indicated that tumors injected with hSef-b DNA exhibit a significantly lower number of Ki-67 positive cells (Fig. 4B). Quantification of the number of Ki67 positive cells indicated that proliferation index was reduced by about 60% (Fig. 4C, p ≤ 0.0001).

The dependence of tumor growth on the development of a neovasculature is a well-established aspect of cancer biology^32^. Moreover, specifically in the case of prostate cancer, angiogenesis plays an important role in its progression^33–35^. Given that hSef-b negatively regulates cellular responses to the angiogenesis promoting factor FGF2, and to NF-κB whose targets are involved in angiogenesis [e.g. VEGF, and MMP-9^36^], we hypothesized that hSef-b expression might affect tumor growth also by inhibiting the process of tumor angiogenesis. This assumption was supported by the observation that tumors which received the empty vector were highly vascularized, whereas intact tumors that received the hSef-b plasmid displayed a significantly reduced number of blood vessels on the tumor surface (Fig. 3C). To further substantiate this observation, we examined the effect of hSef-b on tumor vasculature by evaluating microvessel density (MVD) following immunostaining of tumor sections for an endothelial cell marker CD31. The data clearly show that blood vessels number and size were markedly reduced in tumors injected with the hSef-b DNA (Fig. 4D and E, 56% reduction in microvessel density as compared to control, p ≤ 0.0016).

**Figure 5:**
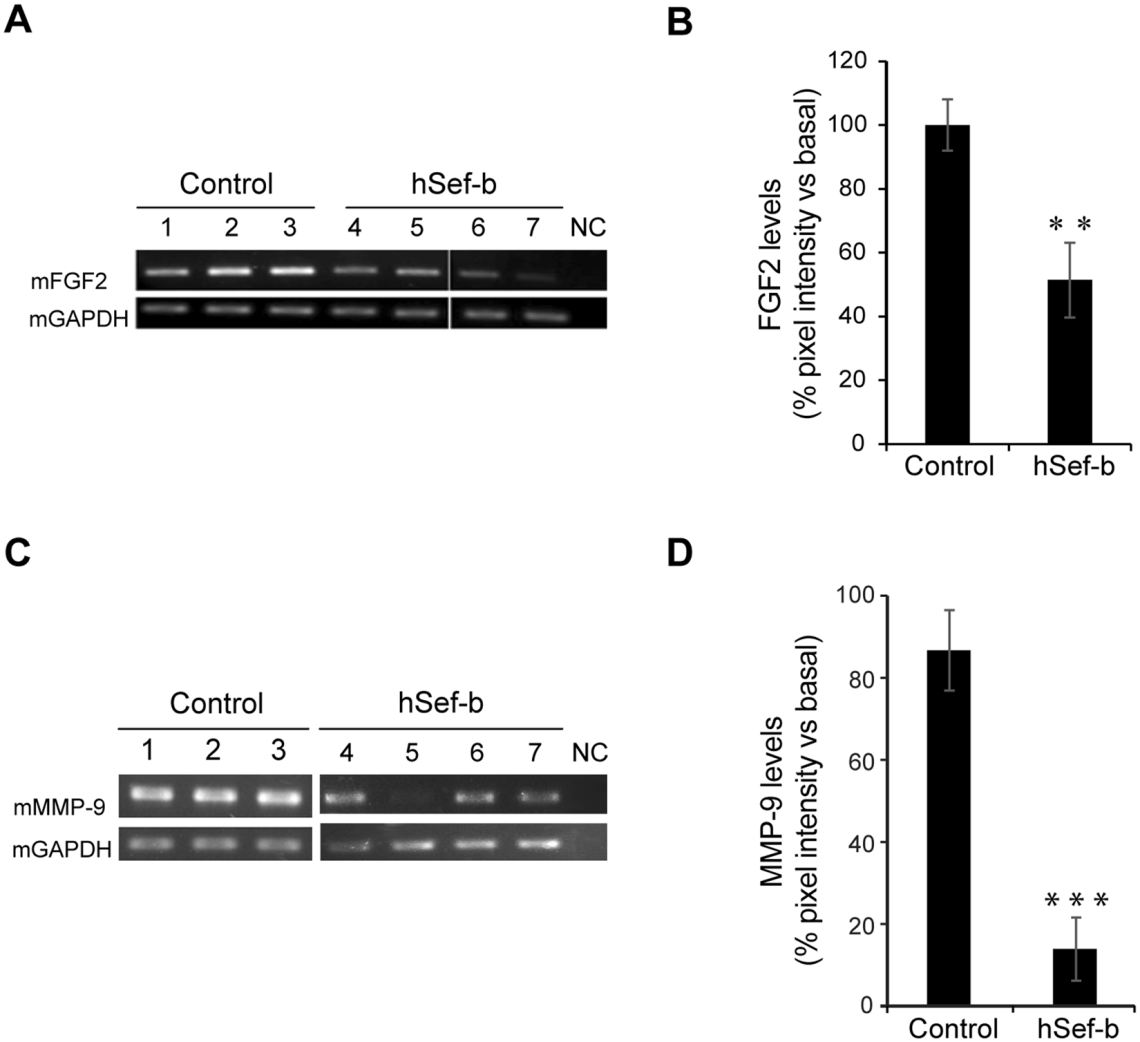
The effect of repeated TUS treatments with hSef-b plasmid on the expression of FGF2 and MMP-9. **(A**,**C**) FGF2/MMP-9 mRNA levels in tumors transfected repeatedly with control (N = 3) or hSef-b construct (N = 4) using TUS. NC denotes negative control were RT-PCR was carried out in the absence of template. (**B**,**D)** Quantification of FGF2 and MMP-9 mRNA expression levels normalized to GAPDH. Quantification was done using TINA software. The mean value from 2 independent experiments is presented (**P ≤ 0.01 for FGF2 and ***P ≤ 0.001 for MMP-9).

To gain further insight into the mechanism by which hSef-b inhibits PCa growth and neovascularization we analyzed the expression levels of FGF2 and matrix metalloproteinase-9 (MMP-9) in tumors transfected with the control (N = 3) or the hSef-b (N = 4) plasmid by RT-PCR. FGF2 and MMP-9 mRNA levels were reduced in all the tumors transfected with the hSef-b vector (Fig. 5, panels A and C). Densitometry analysis of data from two independent experiments indicated that FGF2 levels were reduced by 50–60% (p ≤ 0.01) and MMP-9 levels by 80–85% (p ≤ 0.001) in tumors transfected with the hSef-b plasmid as compared with tumors transfected with the control vector (Fig. 5 panels B and D).

## Discussion

Prostate tumors are the leading cause of death among males in Western countries. Androgen deprivation represents the main mode of treatment of the advanced disease. Unfortunately, tumors become refractory by progressing into androgen independent stage for which effective treatments are not available. In the present work we assessed the potential of the tumor suppressor, hSef-b, a known natural inhibitor of FGF signaling^11^, for prostate cancer gene therapy using a syngeneic FGF-dependent prostate tumor cell line, TRAMP C2, as a model system. *In vitro*, ectopic hSef-b inhibited FGF induced ERK/MAPK reporter activity and suppressed TRAMP C2 growth consistent with its known capacity to inhibit FGF-dependent ERK/MAPK activation and mitogenic activity in NIH 3T3 fibroblasts^10^. Most importantly, we showed here, for the first time, that hSef-b potently attenuated NF-κB nuclear translocation and consequently NF-κB transcriptional activity in TRAMP C2 cells following stimulation with major pro-inflammatory cytokines, TNF and IL-1. hSef-b also inhibited cytokine induced NF-κB activation in human cervical carcinoma cells. Collectively, our findings establish that hSef-b is capable of inhibiting two pathways critical for PCa progression^2–4,36^. Therefore, we tested the efficacy of hSef-b in suppressing prostate tumor growth *in vivo* when delivered into pre-established tumors by TUS. Using this approach, we showed that hSef-b effectively inhibits tumor cell proliferation and discovered that it is also furnished with the capacity to inhibit tumor-angiogenesis.

The potential of different ultrasound modalities for cancer gene therapy applications has been demonstrated by various studies. These studies, however, focused mainly on assessing the efficiency of delivering reporter genes [e.g. eGFP and luciferase^31,37^] rather than efficacy. Furthermore, these studies utilized mostly high-intensity focused ultrasound^38–40^ or high-intensity ultrasound modalities^41,42^ for the transfection process, which are considered destructive to various tissues. In contrast, therapeutic ultrasound, which is considered safe for clinical applications, was applied *in vivo* mainly for the delivery of cDNA to muscles^43,44^, liver^27^, kidney^45^, and the vasculature^24,25,46^. In previous studies, we utilized TUS for the delivery of a plasmid into the nucleus of cells transfected *in vitro* ^26,37^ as well as for the transfection of prostate tumors *in vivo* with a plasmid encoding for an inhibitor of angiogenesis^28^. To the best of our knowledge, the effect of a tumor suppressor gene (TSG) delivery on prostate tumor growth *in vivo*, employing TUS as a mean of DNA delivery, has not been reported.

The efficiency of hSef-b gene delivery into the tumors was tested by co-expressing hSef-b and eGFP from a bicistronic construct where hSef-b/eGFP cDNAs were cloned downstream to CMV promoter and IRES, respectively. We showed that both hSef-b and eGFP were co-expressed in transfected HEK 293 cells, indicating that eGFP could serve as a bona fide reporter for hSef-b expression. A single injection of this plasmid i.t. followed by a single TUS treatment using conditions described by us previously (2 W/cm^2^, 30% duty cycle for 20 min) indicated that eGFP was distributed almost all over the tumor bulk peaking around day 4 and declining by day 7 post DNA transfection. The efficiency of transfection was about 15%, which is slightly better than the efficiency reported previously for TUS mediated transfection without a contrast agent^28^. The reduction in tumor growth following three consecutive treatments with TUS-hSef-b vector was remarkably more significant than what would be expected based on the transfection rate (60% reduction as compared to tumors transfected with the control vector). Moreover, immunohistochemistry has shown that tumors receiving repeated treatments display a significant decrease in the number of Ki67 positive cells (60% reduction, p < 0.0001). Repeated treatments with hSef-b also led to a significant reduction in tumor vascularization (56% reduction, p = 0.0016), a previously unknown function of Sef. One plausible explanation for this apparent discrepancy is that the percentage of cells expressing eGFP was underestimated owing to the fact that efficiency of translation of IRES-linked genes is generally lower as compared to target genes located upstream to IRES^47,48^. Alternatively, expression of reporter genes may not necessarily assure therapeutic efficacy because certain genes may act not only in a cell autonomous but also in a non-cell autonomous (paracrine) manner, which we believe is the situation with Sef. Paracrine effects of hSef-b are strongly supported by our current findings that hSef-b suppresses *in vivo* expression of FGF2 and MMP-9, and the fact that additional secreted factors controlling various aspects of tumorigenesis are targets of the pathways shown here to be inhibited by hSef-b. It is noteworthy that targeting hSef during human tissue regeneration dysregulates the expression of secreted factors involved in the control of cell proliferation, matrix remodeling and angiogenesis (Haddad J., unpublished results).

Neovascularization is essential for the progression of most solid tumors^32,49^. In PCa, microvessel density has been shown to be a predictor of metastasis and survival^33,35^. In addition to regulating cell growth in a cell autonomous and a paracrine manner^50–54^, the NF-κB and the ERK/MAPK pathways also induce the activation of proangiogenic factors such as vascular endothelial growth factor (VEGF), FGF2 and matrix metalloproteinases [MMPs^52,54^]. Hence, blockade of NF-κB was reported to inhibit *in vitro* and *in vivo* expression of vascular endothelial growth factor (VEGF), MMP-9 and interleukin-8 (IL-8) and consequently decreased neoplastic angiogenesis in human prostate cancer cells^52^. ERK/MAPK is known to induce FGF2 through the activation of the Egr-1 transcription factor which is a target of Elk1^55,56^. Thus, the reduced expression of FGF2 and MMP-9 in hSef-b transfected tumors is consistent with hSef-b ability to inhibit the activation of both NF- κB and Elk-1 (see Fig. 1, panels B–E).

In summary, we demonstrated here, for the first time, the efficacy of a non-viral TUS-based hSef-b gene delivery approach for the treatment of prostate cancer tumors. Using this approach, we showed that hSef-b negatively regulates two biological processes essential for tumor progression through its ability to attenuate both FGF and pro-inflammatory cytokine signaling. Most importantly, our results point to the potential therapeutic benefit of restoring hSef-b expression not only for prostate cancer but also for a variety of human carcinoma types where hSef expression is down-regulated.

## Materials and Methods

### Reagents, Antibodies, and Constructs

IL-1α and TNF-α were from Peprotec. FGFs and hSef antibodies were produced as previously described^10,57^. Anti c-Myc-tag (9E10, sc-40), and p65 (sc-372) were from Santa Cruz Biotechnology. FITC-conjugated goat anti-rabbit IgG was from ICN, and rhodamine-red-X-conjugated Affinipure goat anti-mouse IgG was from Jackson Immuno-Research. Anti Ki67 (clone SP6) was from Lab Vision and anti CD-31 was from BD Pharmingen. Myc-tagged hSef-b and hSef-a expression vectors were previously described^10,14^. To monitor the efficiency of DNA delivery into the tumor cells *in vivo*, we generated a bicistronic vector in which a cassette, containing the hSef-b cDNA followed by an internal ribosome entry site (IRES) element and eGFP cDNA, was inserted downstream to the CMV promoter in PLNCX or pCDNA3.1 (designated hSef-b/eGFP vector). IRES-eGFP alone was also cloned into PLNCX to serve as a control vector. The plasmid DNAs were amplified and purified using Qiagen kit. For inducible expression, Myc-tagged hSef-b was cloned in the tetracycline-inducible vector pSG213 (a gift from Pier Paolo Di Fiore, Institute of Molecular Oncology, IFOM, Milan, Italy). All the expression constructs contain the natural translation-initiation codons of each hSef isoform (AUG and CUG for hSef-a and hSef-b, respectively).

### Cell Cultures and Transfections

HeLa and HEK 293 cells were maintained in Dulbecco’s modified Eagle’s medium **(**DMEM) supplemented with 10% Fetal Bovine Serum (FBS). Murine prostate cancer cells derived from transgenic adenocarcinoma of mouse prostate (TRAMP C2) were cultured in DMEM (Gibco laboratories) supplemented with 5% FBS and 5% Nu-Serum (BD Bioscience), 10^−8^ mol/L of dihydrotestosterone (Sigma), and 5 μg/mL of insulin (Sigma). All media were supplemented with penicillin-streptomycin solutions (Biological Industries- Bet Haemek, Israel) and the cultures were grown at 37 °C and 5% CO_2_. For hSef-b inducible expression, TRAMP C2 cells were transfected with pSG213/hSef-b or the empty vector, and marker selected with puromycin (2.5 µg/ml). Colonies of resistant cells that did not express detectable levels of hSef-b protein in the absence of dox were chosen for further analysis. For IF, cells were seeded onto gelatin coated coverslips, and the next day hSef-b expression was induced by adding dox (2 µg/ml) and BSA (0.5%) into TRAMP C2 growth medium for 24 hrs.

Transient transfections in HEK 293 and Hela cells as well as stable transfections in TRAMP C2 cells were performed with DreamFect reagent (OZ Biosciences) as previously described^17^. Transient transfections in TRAMP C2 cells were performed using microporator (Invitrogen) under conditions optimized for TRAMP C2 cells electroporation. Briefly, cells were washed once with complete growth medium lacking antibiotics, then, washed twice with PBS and re-suspended in manufacturer supplied R-Buffer. Electroporation (one pulse at 1300 V for 20 ms) was carried out with 4 × 10^5^ cells and the desired amounts of DNA. Cells were then seeded onto 24 wells for luciferase reporter assay.

### Dual luciferase assay

The assay was performed with either Elk-1 (pSRE) or NF- κB luciferase-reporter plasmid. Cells were transfected with 0.33 µg Luciferase reporter plasmid, 0.033 µg Renilla and 0.5 µg of hSef plasmid (hSef-a or hSef-b) or control empty vector. 16 hours post transfection, cells were left untreated or treated with the indicated ligands for 4 hr (TRAMP C2 cells) or 6 hr (Hela cells). Luciferase activity in cell lysates was measured by using the luciferase assay system (Promega, Madison, WI, USA) in a GLOMAX 20/20 luminometer. Reporter activity was normalized to the activity of the co-expressed Renilla.

### Ultrasound Apparatus and *In vivo* Gene Transfection

The ultrasound apparatus used for all experiments is a therapeutic ultrasound, which operates at a frequency of 1 MHz (UltraMax, XLTEK Canada). Mice, C57BL/6 male 4 to 5 weeks old, were inoculated s.c. in the flank with 2 × 10^6^ TRAMP C2 cells per mouse. When tumors reached ∼100 mm^3^, animals were randomly divided into groups with 5–8 mice in each group, and injected with 100 μg of control or hSef containing plasmid. When indicated, controls also included therapeutic ultrasound alone.

TUS was applied as previously described^31^ and operated at 30% duty cycle, 2 W/cm^2^ for 20 min. The effect of TUS was studied either after one or after repeated cDNA administrations. Mice were sacrificed 3, 4 and 7 days post a single cDNA/TUS application. For the longer term experiments, cDNA/TUS applications were repeated 3 times with weekly intervals and animals were sacrificed 21 days post first application. Subcutaneous tumor growth was measured with a caliper every 2 days during the 21 day period, and tumor volume was calculated as described^58^. Tumors were harvested at the end of the experiment, photographed and weighed. Tumor samples were taken for RT-PCR analysis and the remaining tumors were taken for histological examination and immunohistochemistry. All animal studies were approved by the institutional (Technion) Animal Ethics Committee. All experiments were performed in accordance with relevant guidelines and regulations.

### RNA Preparation and Reverse Transcription-PCR

hSef mRNA expression in transfected TRAMP C2 tumors was evaluated 21 days post-first plasmid DNA delivery/therapeutic ultrasound application using reverse transcription-PCR (RT-PCR). Total RNA was extracted using Tri-Reagent (Sigma), following standard protocols, and 1 µg from each sample was taken for synthesis of cDNA using random primers. PCR was performed with primers specific to hSef-b (5′-CTCTGCTCCGTCTTCTTTAC-3′ and 5′-CTGTTGAGCTGCTTCGGATC- 3′); mouse glyceraldehyde-3- phosphate dehydrogenase (GAPDH) control (5′-GGT GAA GGT CGG AGT CAA CGG A-3′ and 5′-GAG GGA TCT CGC TCC TGG AAG A-3′); mouse FGF2 (5′-ATGGCTGCCAGCGGCATCACCT-3′ and 5′-CCAGTTCGTTTCAGTGCCACATAC-3′). Amplification was performed as previously described^10,59^.

### Histology, Immunohistochemistry, and Immunofluorescence

Harvested tumors were embedded in optimal cutting temperature compound (OCT, Tissue-Tek, Sakura), frozen in liquid nitrogen and stored at −80 °C. Sections (7 μm) from each tumor were stained using H&E. Immunohistochemistry was carried out using Vectastain Elite ABC kit (Vector Laboratories). Primary antibodies include anti-CD31 (1:100; BD Bioscience) for microvessel staining and anti–Ki-67 nuclear antigen (1:100; LabVision) for proliferating cells. Detections were carried out using the 3,3′-diaminobenzidine chromogen (Vector Laboratories) and sections were counterstained with hematoxylin. Negative control slides were obtained by omitting the primary antibody. Microvessel density was assessed according to a method described elsewhere^60^. The percentage of the microvessel areas were determined by LUCIA image analysis software using 10 randomly chosen fields per section in at least 3 sections from 4 different tumors at ×100 magnification. The proliferation index was defined as the percentage of positively stained cells of 100 nuclei from 10 randomly chosen fields at ×200 magnification, as previously described^61^. IF for testing NF-κB(p65) nuclear translocation was performed as previously described^17^. Nuclear staining was done with 10 µM DRAQ5 (Biostatus Limited). Images were examined by using standard fluorescence or confocal microscopy. Transfection efficiency of hSef-b/eGFP plasmid into TRAMP C2 tumors *in vivo* was estimated on tumor tissue sections (20 µm) by comparing the number of eGFP positive cells relative to the total number of cells in a microscopic field stained with Hoechst. Sections were mounted with Fluoromount-G (EMS), and eGFP expression in the tumors was viewed with LSM 510 laser confocal microscope (Carl Zeiss). Transfection efficiency was quantified in three randomly chosen fields per section in at least five sections using Laboratory Universal Computer Image Analyses (LUCIA, Laboratory Imaging, CZ).

### Statistical Analysis

All data are expressed as mean value ± SD or expressed as a percentage relative to control ± SD or SEM as indicated. Statistical differences between treatment groups were determined using Student’s t-test for independent samples and GraphPad Prism 5 software. Statistical significance was defined as P < 0.05.

## Electronic supplementary material

Figure S1
