# Supplementary material for: Delivery of the gene encoding the tumor suppressor Sef into prostate tumors by therapeutic-ultrasound inhibits both tumor angiogenesis and growth
Source: Sci Rep. 2017 Nov 8;7:15060. doi: 10.1038/s41598-017-12408-1 (PMC5678190; doi:10.1038/s41598-017-12408-1)
Supplement: Supplementary file 1 — Figure S1 [file 41598_2017_12408_MOESM1_ESM.pdf]

Supplementary Material For

Delivery of the gene encoding the tumor suppressor Sef into prostate tumors by therapeutic-ultrasound inhibits both tumor angiogenesis and growth

Sabrin Mishel[1,4], Boris Shneyer[1], Lina Korsensky[1], Orit Goldshmidt-Tran[1], Tom Haber[2,3], Marcelle Machluf[2], and Dina Ron[1*]

[1]Department of Biology, Technion, Israel Institute of Technology, Haifa, Israel

[2]Department of Biotechnology and Food Engineering, Technion, Israel Institute of Technology, Haifa, Israel

[3]Present address: Department of Molecular Medicine, Beckman Research Institute, City of Hope, Duarte, CA, USA

[4] Present address: Department of Immunology, Faculty of Medicine, University of Toronto, Program in Genetics and Genome Biology, The Hospital of Sick Children, Toronto, Canada

Corresponding author e-mail address: dinar@ technion.ac.il

Figure S1

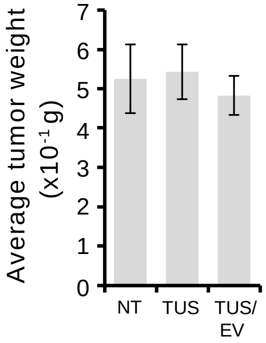

TUS does not facilitate prostate tumor growth *in vivo*. C57BL/6 mice were inoculated s.c. in the flank with $2X10^6$ TRAMP C2 cells per mouse. When tumors reached a palpable size (~ 100 mm^3), animals were either left untreated, treated with TUS alone or TUS following intratumoral injection of control-empty plasmid (N=5 mice in each group). TUS and DNA injection were repeated 3 times in weekly intervals, and tumors were resected one week post last treatment. No significant difference in tumor weight was found between the three groups (p value =0.345).
